# COMP-Angiopoietin1 Potentiates the Effects of Bone Morphogenic Protein-2 on Ischemic Necrosis of the Femoral Head in Rats

**DOI:** 10.1371/journal.pone.0110593

**Published:** 2014-10-17

**Authors:** Lu Zhou, Sun Jung Yoon, Kyu Yun Jang, Young Jae Moon, Sajeev Wagle, Kwang Bok Lee, Byung-Hyun Park, Jung Ryul Kim

**Affiliations:** 1 Departments of Orthopedic Surgery, Chonbuk National University Medical School, Research Institute for Endocrine Sciences and Research Institute of Clinical Medicine, Jeonju, Republic of Korea; 2 Departments of Pathology, Chonbuk National University Medical School, Research Institute for Endocrine Sciences and Research Institute of Clinical Medicine, Jeonju, Republic of Korea; 3 Departments of Biochemistry, Chonbuk National University Medical School, Research Institute for Endocrine Sciences, Jeonju, Republic of Korea; 4 Department of Sports Medicine, Taishan Medical University, Shandong, China; University of California Davis, United States of America

## Abstract

Angiogenesis is considered essential for proper bone regeneration. The purpose of this investigation was to determine if a combined therapy of bone morphogenetic protein-2 (BMP-2) and cartilage oligomeric matrix protein angiopoietin-1 (COMP-Ang1) can potentiate the therapeutic effect of BMP-2 in a rat model of ischemic necrosis of the femoral head (INFH). INFH was surgically induced in the femoral head of rats, and the animals were divided into the following groups: 1) a sham-operated group (sham group), 2) a bovine serum albumin-injected group (BSA group), 3) a BMP-2-injected group (BMP-2 group), and 4) a COMP-Ang1 and BMP-2-injected group (COMP-Ang1 + BMP-2 group) (n = 20/group). Radiologic, histologic, and histomorphometric assessments were performed to assess femoral head morphology, vascular density, and bone resorption activity. Western blots and immunohistochemical staining were performed to evaluate production of BMP-related signaling proteins in C3H10T1/2 cells and tissues. Real-time RT-PCR was performed to investigate expression of the target integrin gene, and the effect of integrin on C3H10T1/2 cells was determined using a cell adhesion assay. Radiographs obtained six weeks after injection revealed better preservation of the architecture of the femoral head in the COMP-Ang1 + BMP-2 group compared with the BSA and BMP-2 groups. Histological findings indicated increased trabecular bone and vascularity and decreased osteoclast bone resorption activity in the COMP-Ang1 + BMP-2 group compared with those in the BSA and BMP-2 groups. The combination of COMP-Ang1 and BMP-2 increased phosphorylation of Smad1/3/5, p38, and Akt. Increased integrin α3 and β1 mRNA expression in the COMP-Ang1 + BMP-2 group promoted cell adhesion. These results suggest that COMP-Ang1 preserved the necrotic femoral head through the potentiation of BMP-2 signaling pathways and angiogenesis. Combination treatment with COMP-Ang1 and BMP-2 may be a clinically useful therapeutic application in INFH.

## Introduction

Ischemic necrosis of the femoral head (INFH) is a relentless process in which ischemia and subsequent defective bone repair lead to collapse of the femoral head [1], [2]. INFH can lead to permanent deformity of the femoral head, severely compromised hip joint longevity, and production of premature end-stage osteoarthritis as early as the third decade of life [3]. Because total hip replacement is unsuitable for the young, active patient population, the goal of early treatment for INFH is to prevent the development of femoral head deformities [4].

The use of biological agents to preserve the femoral head and avoid joint replacement surgery is currently under investigation [5]. Bone morphogenetic protein-2 (BMP-2) promotes the commitment of pluripotent mesenchymal cells to the osteoblast lineage by producing signals that stimulate the specific transcriptional programs required for bone formation [6]. BMP-2 has been shown to stimulate osteoblast recruitment, new bone formation, and angiogenesis under fracture-healing and spinal fusion conditions [7]–[9]. Despite the demonstrated positive effects of BMPs on bone healing, the universal use of recombinant human BMPs is tempered due to high cost and lingering safety concerns including vertebral osteolysis, ectopic bone formation, radiculitis, or cervical soft tissue swelling [10]. Furthermore, BMP-2 is known to stimulate osteoclastogenesis and bone resorption [11], [12]. Vandermeer et al. reported that the local administration of BMP-2 after INFH in a piglet model resulted in increased bone resorption and a similar degree of head deformity compared with animals in the untreated group [13].

The importance of angiogenesis during bone regeneration has been demonstrated in various experimental bone formation models in which stimulation or disruption of normal fracture healing or in vivo bone formation has been evaluated [6]. The therapeutic concepts of angiogenesis and secondary osteogenesis have gained considerable attention in terms of preventing the development of femoral head deformities after ischemic osteonecrosis.

A recombinant COMP-Ang1 protein, a chimeric form of Ang1 containing a minimal coiled-coil domain of the cartilage oligomeric matrix protein sufficient for oligomerization, has been previously synthesized. COMP-Ang1 has potent and stable activity in vascular formation and also overcomes problems associated with aggregation and insolubility of Ang1 over time [14]. COMP-Ang1 has been shown to facilitate necrotic femoral head repair via the enhancement of angiogenesis [5]. Moreover, COMP-Ang1 was shown to synergistically enhance osteoblastic differentiation through the potentiation of BMP signaling pathways and to promote cell adhesion and recruitment via the effects of integrin [15]–[17].

We hypothesized that the ideal repair process following INFH would combine angiogenesis with concomitant stimulation of new bone formation. To our knowledge, the effects of combined therapy consisting of enhancing angiogenesis and osteogenesis in INFH have not been investigated. In this study, we demonstrate that local administration of COMP-Ang1 and BMP-2 enhances bone formation and prevents femoral head deformity through potentiation of BMP-2 signaling pathways and induction of angiogenesis in a surgically induced INFH rat model. Our results provide an effective therapeutic strategy for the treatment of INFH.

## Materials and Methods

### Ethics statement

This research was conducted in accordance with the research and publication guidelines of the Committee on Publication Ethics (COPE), the Declaration of Helsinki, and the Guide for the Care and Use of Laboratory Animals as adopted and promulgated by the United States National Institutes of Health. The Institutional Animal Care and Ethics Committee of Chonbuk National University approved all experimental procedures in this study (Ethics number: CBU2011-0012).

### Animals and surgical procedure

Male Sprague–Dawley rats (six to eight weeks of age, 250–300 g) were used. All rats were handled regularly for at least one week before surgery and were housed in individual cages at 22°C and 50% humidity in controlled rooms having 12 h light/dark cycles. To allow for a 30-min anesthesia period, 150 mg/kg Zoletil (Virbac, Nice, France) was administered intraperitoneally. Ischemic osteonecrosis was induced in the femoral head by surgical application of a tight ligature around the femoral neck, as previously described [5]. The sham operation consisted of opening the capsule but leaving the ligature untied. Two weeks after inducing ischemia, repeat arthrotomy was performed to visualize the femoral head. COMP-Ang1, BMP-2, or BSA was injected through the epiphyseal cartilage by penetrating a 28-G needle to a maximum depth of 3 mm. This depth was used because the diameter of the bony epiphysis of rats ranged from 5 to 6 mm. We considered injection into the epiphysis to be successful when there was no leakage or back flow after intraosseous administration. The rats were assigned to receive bovine serum albumin (BSA group, n = 20), BMP-2 (BMP-2 group, n = 20), or a combination of COMP-Ang1 and BMP-2 (COMP-Ang1 + BMP-2 group, n = 20). Each animal in the BSA group received 100 µg BSA, and each animal in the BMP-2 group received 5.0 µg BMP-2, doses previously shown to be effective in enhancing bone regeneration [18]. Each animal in the COMP-Ang1 + BMP-2 group received 100 µg COMP-Ang1, which is similar to that used in our previous study, and 5.0 µg BMP-2. To minimize the unnecessary use of animals, we did not include a COMP-Ang1-only group. Previously, we reported the effects of COMP-Ang1 using the same ischemia-induction surgery and intraosseous injection of 100 µg COMP-Ang1 on animals in the same age range [5]. Animals were euthanized by exsanguination under sodium pentobarbital anesthesia at eight weeks after inducing INFH.

### Cell culture

C3H10T1/2 cells (ATCC; Manassas, VA, USA) were cultured in α-MEM (Invitrogen, Carlsbad, CA, USA) containing 10% fetal bovine serum (FBS, Invitrogen), 100 U/ml of penicillin, and 100 µg/ml of streptomycin (Invitrogen) in humidified air containing 5% CO2 at 37°C. To induce osteogenic differentiation, C3H10T1/2 cells were treated with osteogenic medium for two weeks, changing the medium twice a week. Osteogenic medium consisted of DMEM supplemented with 10% FCS, 0.2 mM ascorbic acid, 10 mM ß-glycerophosphate, and 0.1 M dexamethasone. BMP-2 was added to the osteogenic medium, and C3H10T1/2 cells were incubated for 14 days. RNA from days 0, 3, 7, and 14 were collected and compared. The expression levels of dentin matrix protein-1 (DMP1), bone sialoprotein (BSP), osteocalcin (OC), aggrecan, Coll21a, and Col10a1 were assayed by real-time RT-PCR to determine if the cells were at the osteogenic or chondrogenic stage of differentiation. Matrix mineralization was detected by staining with Alizarin Red S (AR-S). The stained cultures were photographed, and the AR-S was extracted by 10% (w/v) cetylpyridinium chloride in 10 mM sodium phosphate (pH 7.0) for quantification. The AR-S concentration was determined by measuring the absorbance at 540 nm using a multi-plate spectrophotometer (Bio-Tek instrument, Winooski, VT) and an AR-S standard curve in the same solution.

HEK293 cells (ATCC; Manassas, VA, USA) were cultured in DMEM containing 7.5% inactivated FCS, 50 U penicillin/ml, and 50 g streptomycin sulfate/ml. Human umbilical vein endothelial cells (HUVEC) were cultured in endothelial cell basal medium (EBM; Lonza, Walkersville, MD, USA). We used HEK293 cells as a negative control and HUVEC cells as a positive control for Tie2 expression in real-time RT-PCR.

### Recombinant protein

Recombinant Chinese hamster ovary cells expressing COMP-Ang1 (CAI-2; production rate, ∼30 mg/L) were established as previously described [7]. Recombinant human BMP-2 (rh BMP-2) was obtained from Dae-Woong Pharmaceutical (Seoul, Korea).

### Assessment of bone destruction by MicroCT

**Figure 1:**
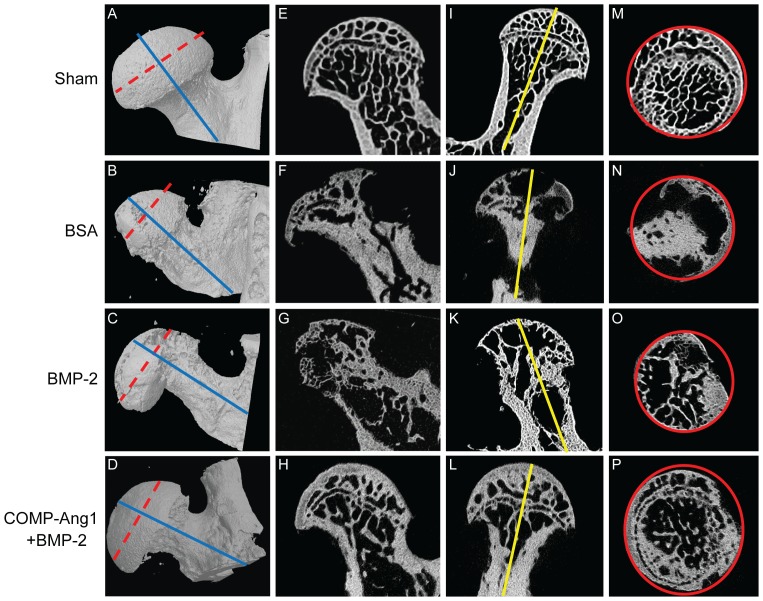
Representative micro-CT scan images of infarcted femoral heads in the BSA, BMP-2, and COMP-Ang1 + BMP-2 groups. Shown are femoral heads obtained from the infarcted area eight weeks after surgery-induced avascular necrosis. Femoral heads showed significant loss of trabecular bone and deformity in the BSA group (B, F, J, N). Femoral heads in the BMP-2 group (C, G, K, O) showed a prominent bone resorption area, but maintained femoral head shape as compared with the BSA group. The trabecular network and femoral head architecture were well preserved in the COMP-Ang1 + BMP-2 group (D, H, L, P). The ROIs (solid red lines) of each group (M, N, O, P) were obtained from the cross-sectional area of the middle of the femoral head (dotted red lines). The sagittal images (I, J, K, L) were formed by the cross-sectional area of the blue lines perpendicular to the dotted red lines, and the coronal images (E, F, G, H) were formed by the cross-sectional area of yellow lines.

A SKYSCAN 1076 Micro-CT unit (Skyscan, Kontich, Belgium) was used to assess bone volume within the defect site. The unit is intended for non-invasive imaging of the internal microstructures of objects with micrometer resolution and consists of a microfocus X-ray source, a precision object manipulator, and high resolution CCD detectors. The source was of the open tube type and the minimum focal spot size was <5 m. The spatial resolution of the micro-CT device can extend up to 4000 × 2600 pixels. In the present study, the X-ray source was set at 75 kV and 100 µA, with a pixel size at 8.8 µm, and 400 projections were acquired over an angular range of 180° (angular step of 0.45°). The image slices were reconstructed using cone-beam reconstruction software based on the Feldkamp algorithm (Dataviewer; Skyscan, Belgium). The area included in the CT scans extended from the upper margin of the epiphysis of the femoral head to femoral neck. A global thresholding algorithm was applied at a constant threshold for all specimens. This threshold was chosen as the intensity (gray value) that corresponded to ∼45% of the average intensity of the intact cortical bone in the specimens. Voxels with intensities higher than the threshold were considered to contain mineralized tissue. A constrained 3D Gaussian filter (filter width = 0.8, filter support = 1 voxel) was used to partially suppress image noise. All aspects of the system were operated using software (Dataviewer; Skyscan, Belgium). On the stacked reconstructed micro-CT cross-section images, manual regions of interest (ROIs) of an irregular anatomical contour were drawn on the transverse image at middle of the femoral head (Figure 1). The volume of interest (VOI) consisted of a stack of ROIs drawn over 52 cross-sections, resulting in a height of 0.45 mm. The VOI included the subchondral trabecular bone, the secondary ossification center, and the growth plate in the ischemic femoral head, excluding the non-ischemic femoral neck and intertrochanteric area distal to the ligation of the femoral neck. The following three-dimensional (3D) morphometric parameters were calculated for the VOI of the femoral head (Skyscan, Kontich, Belgium): bone volume (BV, mm^3^), bone volume fraction (BV/TV, %), trabecular thickness (Tb.Th, μm), trabecular separation (Tb.Sp, μm) and trabecular number (Tb.N, mm^−1^). BV is the 3D of the structure segmented as bone, and BV/TV is the ratio of the segmented bone volume to the total volume of the region of interest. Tb.Th is the mean thickness of the trabeculae, Tb.Sp is the mean distance between trabeculae, and Tb.N is the average number of trabeculae present per unit length. Tb.Th and Tb.Sp were assessed using direct 3D methods, and Tb.N was calculated using the formula Tb.N  =  (BV/TV)/Tb.Th.

### Histological evaluation

The resected rat femurs were fixed in 10% neutral buffered formalin. After fixation, tissues were decalcified in 10% EDTA for ten days or in rapid decalcifying solution (Calci-Clear Rapid, National Diagnostics, Atlanta, GA, USA) for 12 h and then embedded in paraffin. Tissues were longitudinally sectioned, and the histological sections were obtained at the midline of the femoral head, the most representative area of the femoral head. The histologic sections were stained with hematoxylin and eosin (H&E), Safranin-O, or TRAP (Sigma-Aldrich, MA, USA) for light microscopic analysis. The degree of damage of the femoral head was scored from zero to five as follows: 0, no damage; 1, mild damage with maintained articular cartilage and architecture of the femoral head; 2, destruction of the articular cartilage and collapse of the secondary ossification center but with destruction of less than one-third of the femoral head; 3; destruction of between one-third and two-thirds of the femoral head; 4, destruction of more than two-thirds of the femoral head; 5, complete or near-complete destruction of the femoral head.

### Immunohistochemical staining

After deparaffinizing, sections were treated with sodium citrate buffer in a pressure cooker for 12 min to facilitate antigen retrieval. After blocking endogenous peroxidase, sections were incubated with Protein Block Serum-Free (DAKO, Carpentaria, CA) at room temperature for 10 min to prevent nonspecific staining and then with antibody for factor VIII-related antigen (1∶50, Chemicon, Temecula, CA, USA), CD68 (clone ED1, 1∶100, AbD Serotec, Oxford, UK), phosphor-Smad1/5/8 (1∶100, Cell Signaling, Beverly, MA, USA), or phosphor-Smad2/3 (1∶50, Santa Cruz Biotech, Santa Cruz, CA, USA) for 2 h at room temperature. Peroxidase activity was detected using the enzyme substrate 3-amino-9-ethylcarbazole. Negative control sections were treated in an identical manner except that they were incubated with normal mouse IgG (1∶100, Santa Cruz Biotech, Santa Cruz, CA, USA) instead of specific primary antibody.

### Vascular density measurements and evaluation of bone resorption activity

To evaluate vascular density, factor VIII-related antigen-stained images were then acquired at ×200 magnification using a Nikon ECLIPSE E600 microscope with a 20× objective lens (Plan Fluor20/0.50NA, Nikon), a digital camera (Nikon DXm1200F), and appropriate software (Nikon ACT-1 2.62). We obtained two images of the most vascularized area of the femoral head from each rat without knowledge of the experimental group.

We acquired six images from six rats in each group (sham group, BSA group, BMP-2 group, and COMP-Ang1 + BMP-2 group). The area of one image was 0.33 mm^2^. Vascular densities were determined using an image analysis system (analySIS, Soft Imaging Systems, Germany). Vascular density was calculated as (Factor VIII stained vascular area/total image area in ×200 magnification) ×100 (%).

To investigate osteoclast bone resorption activity in the femoral head, TRAP- or CD68-positive multinucleated cells containing three or more nuclei were counted at highest numbered five high-power fields (x400 magnification) in each femoral head. The area of one high-power field was 0.238 mm^2^. Therefore, the total area analyzed per case was 1.188 mm^2^. Six rats from each group were evaluated.

### Western blot analysis

The entire femoral head was harvested from five rats in each group and decalcified in rapid decalcifying solution (Calci-Clear Rapid) for 12 h. Soft tissue were trimmed from the specimens, washed with phosphate buffered saline to removed contaminants, and then ground to a fine powder using a manual biopulverizer cooled in liquid nitrogen. Total cell extracts were generated by harvesting in a lysis buffer (Cell Signaling, Beverly, MA, USA) and then centrifugation at 12,000×g for 15 min at 4°C. Quantification of total protein was performed with BCA protein assay reagent (Bio-Rad Laboratories, Hercules, CA, USA). Proteins were resolved on a 10% SDS-PAGE gel and transferred to a PVDF membrane. After blocking in 5% milk in Tris-buffered saline with 0.1% Tween-20 (TBST), the membrane was incubated with primary antibodies specific for phospho-Smad, phospho-p38, phospho-Akt, total Smad, p38, and Akt (Cell signaling). After washing, the blots were incubated with secondary antibody diluted 1∶5,000 in TBS-T at room temperature for 1 h. Signals were detected by an enhanced chemiluminescence (ECL) reagent (Santa Cruz Biotech, CA, USA), according to the manufacturer's instructions. Densitometric analysis was conducted directly from the blotted membrane using the LAS-3000 luminoimage analyzer system (Fujifilm, Tokyo, Japan). The relative phosphorylation level of each protein was calculated as the ratio of phosphorylated to total protein levels. At least five samples from each group were used.

### RNA preparation

A subset of rats was sacrificed four weeks after surgery. Five rats from each group were evaluated. Total RNA was prepared from the entire mass of the regenerated bone using Trizol reagent (Invitrogen, Carlsbad, CA, USA). Harvesting was performed with the distractor in place in order to accomplish tissue retrieval with precision and accuracy. All extraneous soft tissue was cleaned and the tissue was snap frozen in liquid nitrogen. Total RNA from HEK293, C3H10T1/2 and HUVEC cells were harvested using Trizol reagent. At least five samples from each group were analyzed.

### Real-time RT-PCR

RNA prepared from distraction tissues was precipitated with isopropanol and dissolved in DEPC-treated distilled water. Total RNA (500 ng) was treated with RNase-free DNase (Invitrogen) and first-strand cDNA was generated using random hexamer primers provided in the first-strand cDNA synthesis kit according the manufacturer's protocol (Applied Biosystems, Foster City, CA, USA). Specific primers for each gene were designed (Table 1) using Primer Express software (Applied Biosystems). The GAPDH gene sequence was used as an invariant control. The real-time RT-PCR reaction mixture consisted of 10 ng reverse transcribed total RNA, 2.0 nM forward and reverse primers, and 5 × PCR master mixture in a final volume of 10 µl. The PCR reaction was carried out in 384-well plates using the ABI Prism 7900HT Sequence Detection System (Applied Biosystems). All experiments were performed via triplicate cDNA syntheses from the same total RNA (n = 5, each group).

**Table 1:** Sequences and accession numbers for forward (FOR) and reverse (REV) primers used in real-time RT-PCR.

Gene	Sequences for primers	Accession No.
Runx2	FOR: TCCCCGGGAACCAAGAAG	**NM_053470.1**
	REV: GGTCAGAGAACAAACTAGGTTTAGA	
BSP	FOR: CCGGCCACGCTACTTTCTT	**J04215.1**
	REV: TGGACTGGAAACCGTTTCAGA	
Osteopontin	FOR: CTGGCAGTGGTTTGCTTTTG	**AB001382**
	REV: CCACTTTCACCGGGAGACA	
Type 1 collagen	FOR: CGATGGCGTGCTATGCAA	**Z78279**
	REV: TCGCCCTCCCGTTTTTG	
Osterix	FOR: CATCTAACAGGAGGATTTTGGTTTG	**NM_053470**
	REV: AAGCCTTTGCCCACCTACTTTT	
Osteocalcin	FOR: AAGCCCAGCGACTCTGAGTCT	**NM_013414.1**
	REV: AGGTAGCGCCGGAGTCTATTC	
SMAD1	FOR: CTTCTTAGTTTGAAGTCCAGAAGAG	**NM_005900**
	REV: CCACTCGTGCTCCCACA	
SMAD2	FOR: CACGCTAGGAAAACAGCCTC	**NM_005901**
	REV: TCGGAAGAGGAAGGAACAAA	
Akt1	FOR: TGAAGGTGCCATCATTCTTG	**NM_005163**
	REV: ATGAGCGACGTGGCTATTGT	
MAPK14(p38a)	FOR: TGGAGAGCTTCTTCACTGCC	**NM_139013**
	REV: CGAGCGTTACCAGAACCTGT	
TEK(Tie2)	FOR: TTTCGGCATCAGACACAAGA	**NM_013690**
	REV: CCGGCTTAGTTCTCTGTGGA	
ITGA3 (integrin α3)	FOR: CTGGCAGTGGTTTGCTTTTG	**NM_013565**
	REV: AGACTGAGCGACAACAGCG	
ITGB1 (integrin β1)	FOR: CAGTCCAATCCAGAAAATTGG	**NM_002211**
	REV: GAGTCGCGGAACAGCAG	
Ets-1	FOR: TGAGGGGACACAGGTACACA	**NM_011808**
	REV: ATCTCGAGCTTTTCCCTTCC	
GAPD	FOR: AATGAAGGGGTCATTGATGG	**NM_002046**
	REV: AAGGTGAAGGTCGGAGTCAA	
Dmp1	FOR: CCCAAAGGAACACAAGGAGA	**NM_016779**
	REV: TTCGCTGAGGTTTTGACCTT	
BSP	FOR: TGAAGAGTCACTGCCTCCCT	**NM_008318**
	REV: GTCTTTAAGTACCGGCCACG	
OC (bglap1)	FOR: CAAGCAGGGTTAAGCTCACA	**NM_007541**
	REV: GGTAGTGAACAGACTCCGGC	
Aggrecan	FOR: CCCTCAGAGTCACAAAGACCA	**NM_007424**
	REV: TTCGCAGGGATAAAGGACTG	
Col2a1	FOR: GCAAGATGAGGGCTTCCATA	**NM_031163**
	REV: CTACGGTGTCAGGGCCAG	
Col10a1	FOR: ACCAGGAATGCCTTGTTCTC	**NM_009925**
	REV: CATAAAGGGCCCACTTGCTA	

### Cell adhesion assay

Ninety-six well plates were precoated with 20 mg/ml fibronectin (Sigma-Aldrich, MA, USA). C3H10T1/2 cells were trypsinized and incubated in DMEM without serum for 45 or 30 minutes, respectively. The unattached cells were washed away, and the remaining attached cells were fixed with 70% ethanol and stained with 0.1% crystal violet in 20% ethanol. Cells were then destained in 10% acetic acid, and the absorbance value (OD) was measured at 597 nm. Wells coated with bovine serum albumin served as the negative control. All adhesion experiments were performed in triplicate wells and were repeated at least three times.

### Statistical analysis

Statistical analyses were performed by one-way ANOVA followed by a LSD post-hoc test. Total bone volumes were compared across groups using a Kruskal-Wallis test (ANOVA by ranks). Data are expressed as the mean ± SEM. Differences with p values <0.05 were considered statistically significant.

## Results

All rats survived the surgery and returned to normal activity within 24 h. Six rats died during the postoperative period, including two from the sham group, one each from the BSA and BMP-2 groups, and two from the COMP-Ang1 + BMP-2 group. The remaining 74 animals experienced an uncomplicated postoperative course until eight weeks post-surgery. The hips of all animals, including those in the sham group, were found to be dislocated. This dislocation occurred soon after the first surgery. Consequently, all rats walked with a limp.

BMPs are well known to induce heterotopic ossification. Radiographs performed at the time of animal sacrifice showed multiple round ossifications around the hip capsule in the BMP-2 and COMP-Ang1+ BMP-2 groups, which were not observed in the other groups.

### Micro-CT assessment of ischemic osteonecrosis

Micro-CT analysis was performed to provide both a structural and quantitative assessment of mineralized skeletal tissue formation at the osteonecrotic site. Femoral heads in the sham group animals did not develop osteonecrosis (Figure 1 A, E, I, M). Femoral heads in the BSA group animals (Figure 1 B, F, J, N) showed significant loss of trabecular bone and less preservation of femoral head architecture. Femoral heads in the BMP-2 group animals (Figure 1 C, G, K, O) had preserved femoral head architecture, but showed a prominent bone resorption area. In contrast, the trabecular network and femoral head architecture were relatively well preserved in the COMP-Ang1 + BMP-2 group (Figure 1 D, H, L, P). Micro-CT findings were quantified by measuring bone volume, trabecular number, and trabecular thickness (Table 2). The BV in the areas undergoing repair eight weeks after surgery was significantly higher in the COMP-Ang1 + BMP-2 group compared to the BSA and BMP-2 groups. The mean BV/TV values and trabecular numbers were significantly lower in the femoral heads of the BSA and BMP-2 groups than in those of the sham group, whereas the COMP-Ang1 + BMP-2 group had bone masses and a microarchitecture that were similar to those of the sham group. In addition, trabecular number and thickness were significantly higher and trabecular separation was significantly lower in the COMP-Ang1 + BMP-2 group than in the BSA or BMP-2 groups.

**Table 2:** Effect of COMP-Ang1 combined BMP-2 on ischemic osteonecrosis in femoral heads as assessed byMicro-CT.

	Sham	BSA	BMP-2	BMP-2+ COMP-Ang1
BV(mm^3^)	10.4±3.3	4.5±2.15^a^	5.9±1.9^b^	9.9±1.0^c,d,e^
BV/TV(%)	52.0±6.5	24.5±3.1^a^	35.8±7.4^b^	61.9±5.6^c,d,e^
Tb.N(mm^−1^)	2.1±0.4	0.9±0.4^a^	1.4±0.06^b^	3.2±0.6^c,d,e^
Tb.Sp(μm)	239.1±0.3	735.4±0.3^a^	459.1±0.5^b^	129.2±0.9^c,d,e^
Tb.Th (μm)	36.7±0.7	83.2±0.9^a^	62.6±0.4^b^	92.0±0.5^c,d,e^

All measurements are expressed as the mean ± SEM. BV, bone volume; TV, tissue volume; Tb.N, trabecular number; Tb.Sp, trabecular separation; Tb.Th, trabecular thickness.

a , p<0.01 vs. Sham; ^b^, p<0.01 vs. Sham; ^c^, p<0.05 vs. Sham; ^d^, p<0.01 vs. BSA; ^e^, p<0.01 vs. BMP-2.

### Histologic assessments

**Figure 2:**
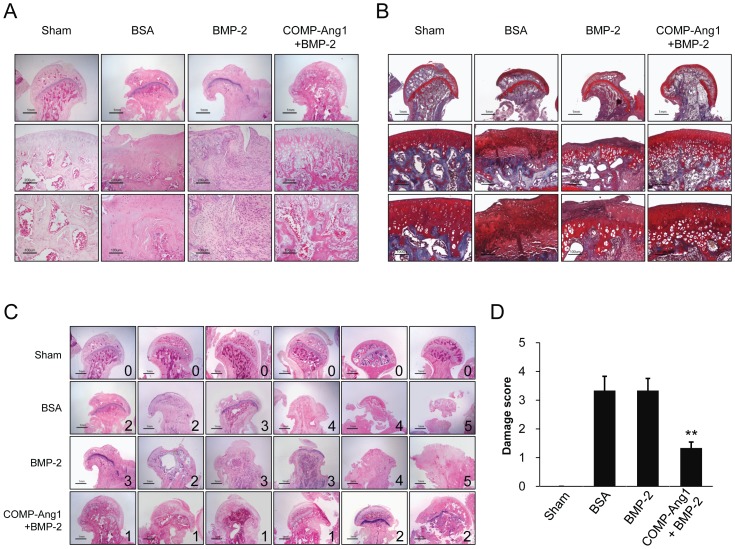
Histologic findings of sham and infarcted femoral heads. (A and B) As compared with the sham-operated group, bone marrow of the femoral heads in the BSA group was necrotic and most of the articular cartilage had been destroyed and replaced by fibrous tissue, as seen on H&E staining (A) and Safranin-O staining (B). Femoral heads in the BMP-2 group showed more obvious deformity and more damaged articular cartilage compared with the sham and COMP-Ang1 + BMP-2 groups. Femoral heads in the COMP-Ang1 + BMP-2 group exhibited relatively well-preserved morphologies. (C and D) The degree of damage to the femoral head was scored from zero to five. The number in each image is the damage score. Femoral heads of the BSA and BMP-2 group had higher damage scores. Damage scores were significantly lower in the COMP-Ang1 + BMP-2 group compared with BSA and BMP-2 groups. **p<0.01 vs. BSA or BMP-2.

Histologically, the femoral heads of the sham group animals were intact and had a smooth articular surface and a well-preserved secondary ossification center (Figures 2A and B). In contrast, femoral heads in the BSA and BMP-2 groups showed more obvious deformities compared with the sham group, and the epiphyseal cartilage surfaces were irregular and partly replaced by fibrous tissue. Especially, the femoral heads of BMP-2 group rats were more damaged and smaller than those in the BSA group. The femoral heads of the BMP-2 group rats were compressed, adhered to adjacent soft tissue, and the secondary ossification center was replaced by fibrous tissue. Femoral heads of BSA and BMP-2 group rats had a higher damage score than that of the sham or COMP-Ang1 + BMP-2 group rats (Figures 2C and D). However, the femoral heads of the COMP-Ang1 + BMP-2 group rats were relatively well preserved compared to those in the BSA or BMP-2 groups. The damage score was significantly lower in the COMP-Ang1 + BMP-2 group compared to the BSA and BMP-2 groups (p <0.01).

The femoral head of the best example from among the COMP-Ang1 + BMP-2 group rats showed a preserved femoral head with smooth articular cartilage and intact bone marrow at the secondary ossification center (Figures 2A and B). The bone in the secondary ossification center was rimmed by osteoblasts with well-formed blood vessels in the bone marrow (Figure 2A).

### Enhanced vascularity by COMP-Ang1 and BMP-2

**Figure 3:**
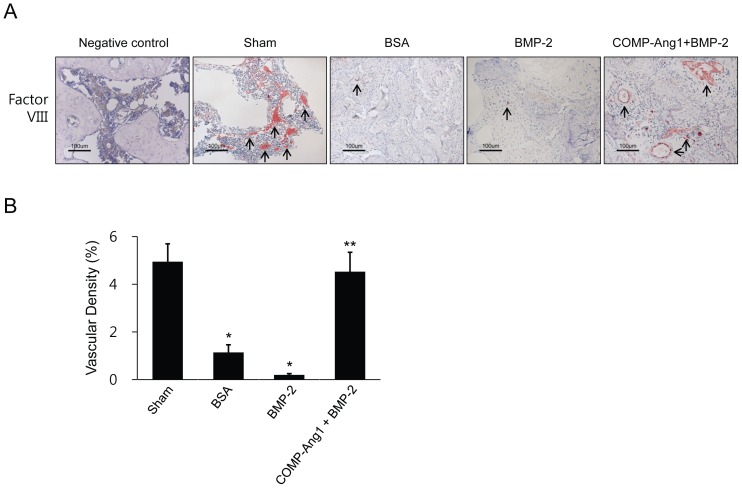
Immunohistochemical staining for factor VIII-related antigen and vascular densities in infarcted femoral heads. (A) Immunohistochemical staining for factor VIII-related antigen in the sham, BSA, BMP-2, and COMP-Ang1 + BMP-2 groups. (B) Vascular densities were measured in six rats from each group and two images were taken (magnification ×200) of each femoral head. The number of factor VIII-related antigen-positive vessels was significantly higher in the COMP-Ang1 + BMP-2 group than in the BSA and BMP-2 groups. Vascular density in each image was calculated as follows: vascular area stained by antibody for factor VIII-related antigen/total area of each image × 100 (%). Data are expressed as the mean ± SEM (n =  6 per group). *p<0.01 vs. sham; **p<0.01 vs. BSA or BMP.

Angiogenesis is known to prevent femoral necrosis, and COMP-Ang1 is considered an angiogenic factor that functions through activation of the Tie2 receptor. Here, to confirm the effects of COMP-Ang1 on vascularity, femoral heads were retrieved, sectioned, and immunostained using antibody against factor VIII-related antigen (Figure 3A). Elevated vessel densities in the COMP-Ang1 + BMP-2 group specimens were confirmed by morphometric analysis of vessels in the secondary ossification center of infarcted femoral heads. As shown in Figure 3B, vascular densities in the BSA and BMP-2 groups were significantly lower than in the sham group (p <0.001); however, the vascular density of the COMP-Ang1 + BMP-2 group was significantly higher compared with that of the BMP-2 group (p <0.001). Vascular densities within the COMP-Ang1 + BMP-2 group were approximately four times higher than those of the BSA group and almost ten times higher than in the BMP-2 group. These differences among the BSA, BMP-2, and COMP-Ang1 + BMP-2 groups paralleled the radiological and histological findings.

### Osteoclast bone resorption is attenuated by COMP-Ang1

**Figure 4:**
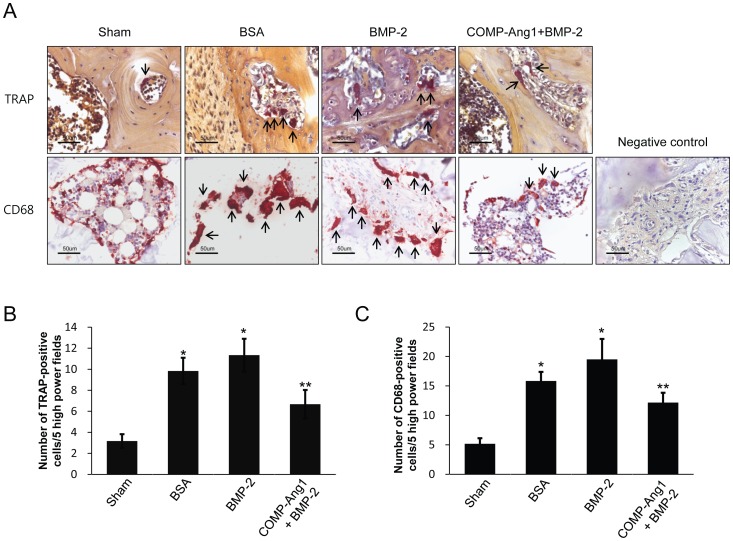
TRAP staining and immunohistochemical staining for CD68 in the femoral head. (A) TRAP and immunohistochemical staining for CD68 in the sham, BSA, BMP-2, and COMP-Ang1 + BMP-2 groups. (B and C) To evaluate osteoclast bone resorption activity, the number of TRAP- or CD68-positive osteoclasts was counted in five high-power fields (x400 magnification) of each femoral head. Arrows indicate TRAP- or CD68-positive osteoclasts. The number of TRAP- or CD68-positive osteoclasts was significantly higher in the BSA and BMP-2 groups than in the sham group. The number of TRAP- or CD68-positive osteoclasts in the COMP-Ang1 + BMP-2 group was higher than in the sham group, but significantly lower than in the BMP-2 group. Data are expressed as the mean SEM (n =  6 per group). *p<0.05 vs. sham; **p<0.05 vs. BSA.

Bone resorption by osteoclasts is important for bone formation and maintenance of bone mass. Therefore, we evaluated osteoclasts using TRAP staining and immunostaining for CD68 (clone ED1). As shown in Figure 4, the number of TRAP- or CD68-positive osteoclasts was significantly higher in the BSA and BMP-2 groups than in the sham group. However, the increased bone resorption activity of the ischemic femoral head, particularly in the BMP group, was attenuated by COMP-Ang1. The number of TRAP- or CD68-positive osteoclasts in the COMP-Ang1 + BMP-2 group was significantly lower than that in the BMP-2 group (p <0.05) (Figures 4B and C).

### Real-time RT-PCR analysis of genes related to osteogenesis

**Figure 5:**
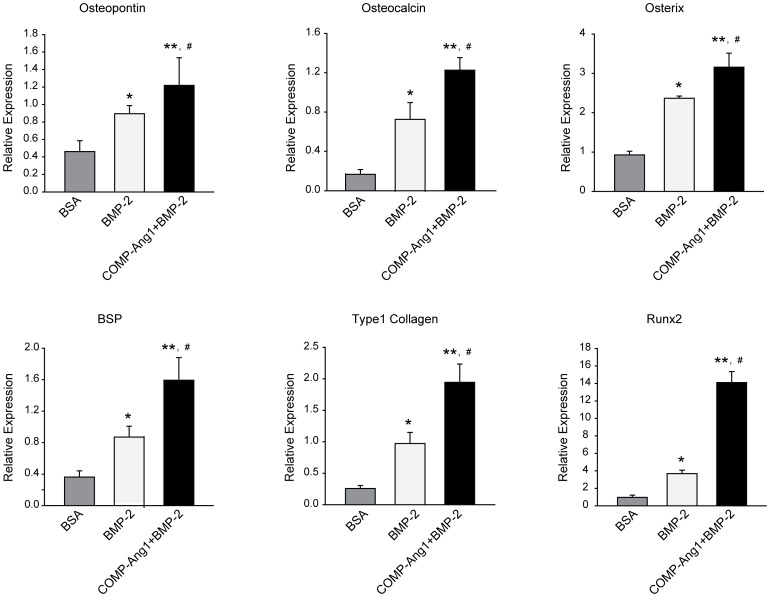
Real-time RT-PCR analyses of genes related to osteogenesis. Tissues were obtained from defect sites three weeks after inducing vascular necrosis. Real-time RT-PCR analyses for osteogenic genes were performed as described in the Materials and Methods. Each value is the mean ± SEM of six to nine samples. *p<0.05 vs. BSA; **p<0.01 vs. BSA; ^#^p<0.01 vs. BMP-2.

We isolated RNA from the femoral head necrosis area and examined the expression levels of several osteogenic genes (Figure 5). The assessed genes included those encoding runt-related transcription factor 2 (Runx2), bone sialoprotein (BSP), type 1 collagen, osteopontin, and osterix. mRNA levels for all osteogenic genes were significantly up-regulated in the BMP-2 group. The COMP-Ang1 + BMP-2 group showed an additional increase in gene expression compared with the BMP-2 group.

### Effect of COMP-Ang1 on BMP-2 signaling pathways in vivo

**Figure 6:**
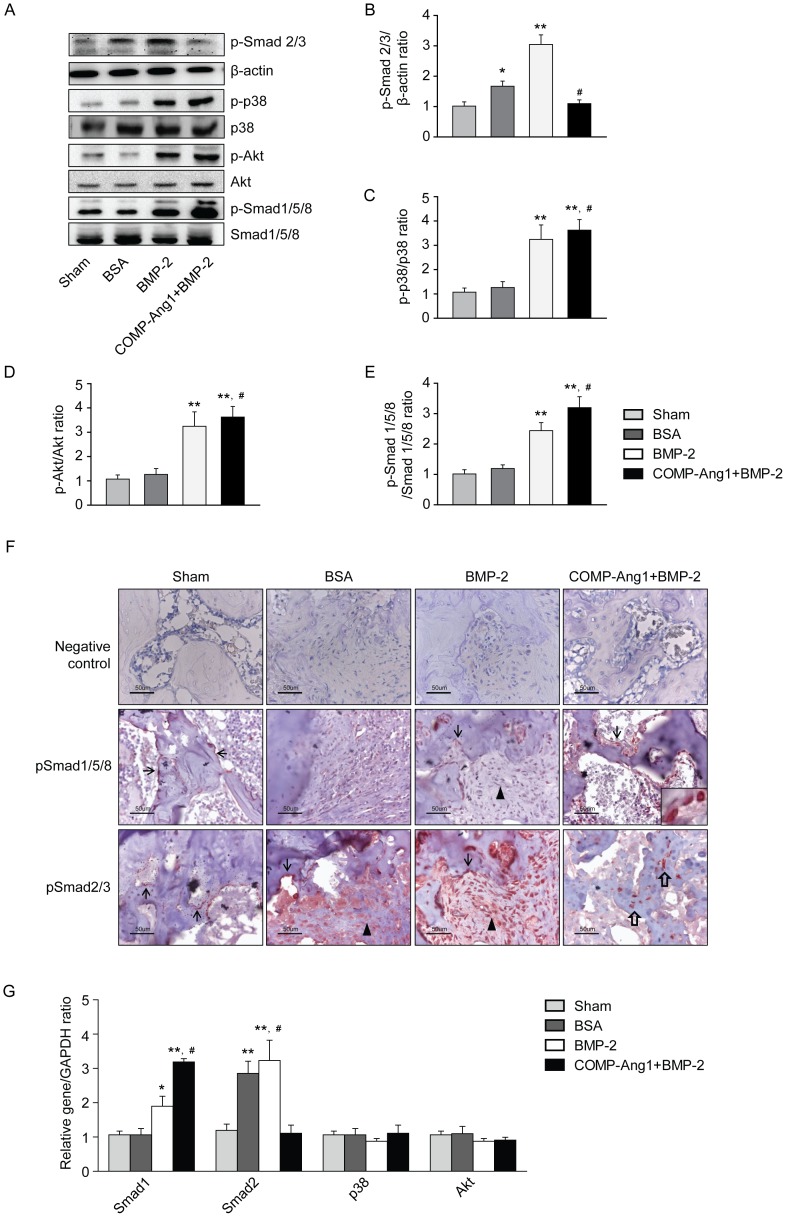
Western blot analysis and immunohistochemical staining of infarcted femoral heads eight weeks after BMP-2 and COMP-Ang1 treatment. Femoral head tissue obtained from defect sites was ground and extracted with protein lysis buffer. Western blot analysis (A) revealed TGF-β related protein expression in the defect sites of the sham, BSA, BMP-2, and COMP-Ang1 + BMP-2 groups, respectively. (B-E) The protein content was calculated by gray scale. (F) Consistent with the results of Western blotting, immunohistochemical expression of phosphor-Smad1/5/8 was stronger in COMP-Ang1 + BMP-2 group osteoblasts (insert). Phosphor-Smad1/5/8 was weakly expressed in the sham, BSA, and BMP-2 group osteoblasts (arrows). In contrast, phosphor-Smad2/3 was strongly expressed in the osteoblasts (arrows) and fibroblasts (arrow heads) of the BMP-2 and BSA groups. Phosphor-Smad2/3 was weakly expressed in the osteocytes (empty arrows) of the COMP-Ang1 + BMP-2 group. (G) BMP signaling-related genes were analyzed by real-time RT-PCR. Data are expressed as the mean SEM (n = 5 per group). *p<0.05 vs. sham; **p<0.01 vs. sham; ^#^p<0.01 vs. BMP-2.

To investigate whether COMP-Ang1 promotes the BMP-2 signaling pathway, the related BMP-2-dependent proteins were examined by Western blot analysis (Figure 6). Compared with the BSA group, phosphorylation of MAPK and Smad1/3/5 was significantly increased in the BMP-2 and COMP-Ang1 + BMP-2 groups. In the COMP-Ang1 + BMP-2 group, p38, Akt, and Smad1/5/8 phosphorylation increased by 1.3-, 1.2-, and 1.4-fold, respectively, relative to the BMP-2 group, whereas phosphorylation of Smad2/3 was decreased 2.5-fold in the COMP-Ang1 + BMP-2 group compared with the BMP-2 group. Immunohistochemical expression of phosphor-Smad1/5/8 and phosphor-Smad2/3 was consistent with the results of Western blot analysis. Phosphor-Smad1/5/8 was strongly expressed in the osteoblasts of the COMP-Ang1 + BMP-2 group osteoblasts,. The while these levels were expression of phosphor-Smad1/5/8 was weak in the osteoblasts of the sham, BSA, and BMP-2 group osteoblastss. In contrast, the expression of phosphor-Smad2/3 was stronger in the BMP-2 and BSA groups and that expressed in the osteoblasts and fibroblasts. In the COMP-Ang1 + BMP-2 group, phosphor-Smad2/3 was weakly expressed in osteocytes (Figure 6 F). Real-time RT-PCR was performed to investigate the mRNA levels of related signaling genes. Smad1 and Smad2 levels were consistent with the Western blot data. Smad1 expression was increased in both BMP and COMP-Ang1 + BMP-2 groups, with relatively higher expression in the COMP-Ang1 + BMP-2 group than in the BMP group, whereas Smad2 was more highly expressed in the BMP-2 group than in the COMP-Ang1 + BMP-2 group. Interestingly, p38 and Akt levels were similar in all groups, which suggests that protein modification occurred after gene transcription (Figure 6 G).

### COMP-Ang1 enhances BMP-2 signaling pathways in vitro

Treatment with BMP-2 increased the expression levels of osteogenic markers (DMP1, BSP, and OC) with time, whereas the expression of chondrogenic markers (aggrecan, Coll21a, and Col10a1) increased until day 3 and then decreased. C3H10T1/2 cells induced with BMP-2 showed a sequential pattern of chondrogenic differentiation followed by osteogenic differentiation (Figure S1 A). Alizarin Red S staining results showed that BMP-2 treatment induced matrix mineralization. The addition of COMP-Ang1 to the BMP-2 treatment synergistically enhanced BMP-2-induced mineralization (Figure S1 B).

**Figure 7:**
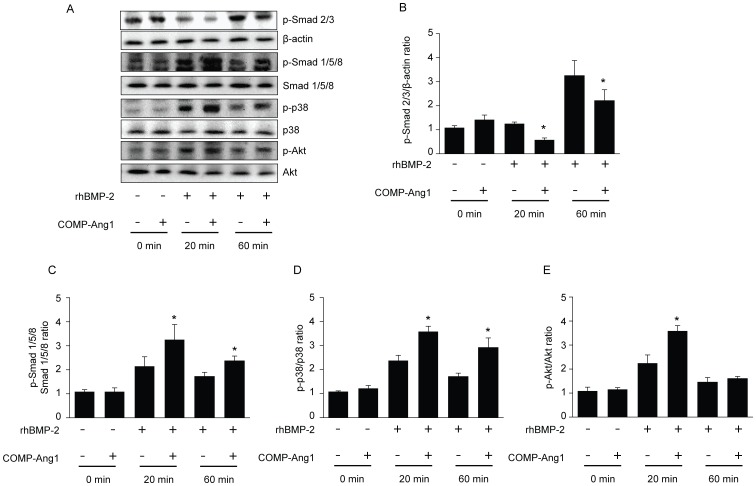
Western blot analysis of osteoblast cells after BMP and COMP-Ang1 treatment. COMP-Ang1 affects BMP2-dependent phosphorylation of Smad, p38, and Akt. (A) C3H10T1/2 cells were treated with rhBMP2 (50 ng/ml) and/or COMP-Ang1 (300 ng/ml) for 20 min or 1 h. (B-E) Quantitative analysis of the phosphorylation of Smad1/5/8, p38, and Akt. Protein content was calculated based on gray scale. Data are expressed as the mean ± SEM (n = 5 per group). ^*^p<0.05 vs.BMP-2.

To further investigate whether COMP-Ang1 influences BMP-2 downstream pathways, we examined the BMP-2 signaling pathways in C3H10T1/2 cells. As shown in Figure 7, BMP-2 stimulated phosphorylation of Smad1/5/8, p38, and Akt by approximately 2-, 2.3-, and 2.3-fold relative to control cells, respectively, at 20 min. The combined COMP-Ang1 + BMP-2 group demonstrated increased Smad1/5/8, p38, and Akt phosphorylation at approximately 3.2-, 3.5-, and 3.7-fold relative to control cells, respectively, at 20 min. In contrast, COMP-Ang1 decreased phosphorylation of Smad2/3, a downstream protein in the transforming growth factor-β (TGF-β) pathway. Our results indicate that the results of 20 min in vitro observation are consistent with in vivo Western blot data. In addition, the COMP-Ang1 + BMP-2 group showed increased Smad1/5/8, p38, and Akt phosphorylation at approximately 2.4-, 2.8-, and 1.5-fold relative to control cells, respectively, at 1 h. Enhancement of BMP-2 by COMP-Ang1 induced phosphorylation that was sustained for up to 1 h. These results suggest that COMP-Ang1 is a positive stimulator of BMP-2 signaling, including the Smad1/5/8, Akt, and p38 pathways, which enhances BMP-2-induced bone formation.

### COMP-Ang1 impacts cell adhesion ability on osteoblast cells

**Figure 8:**
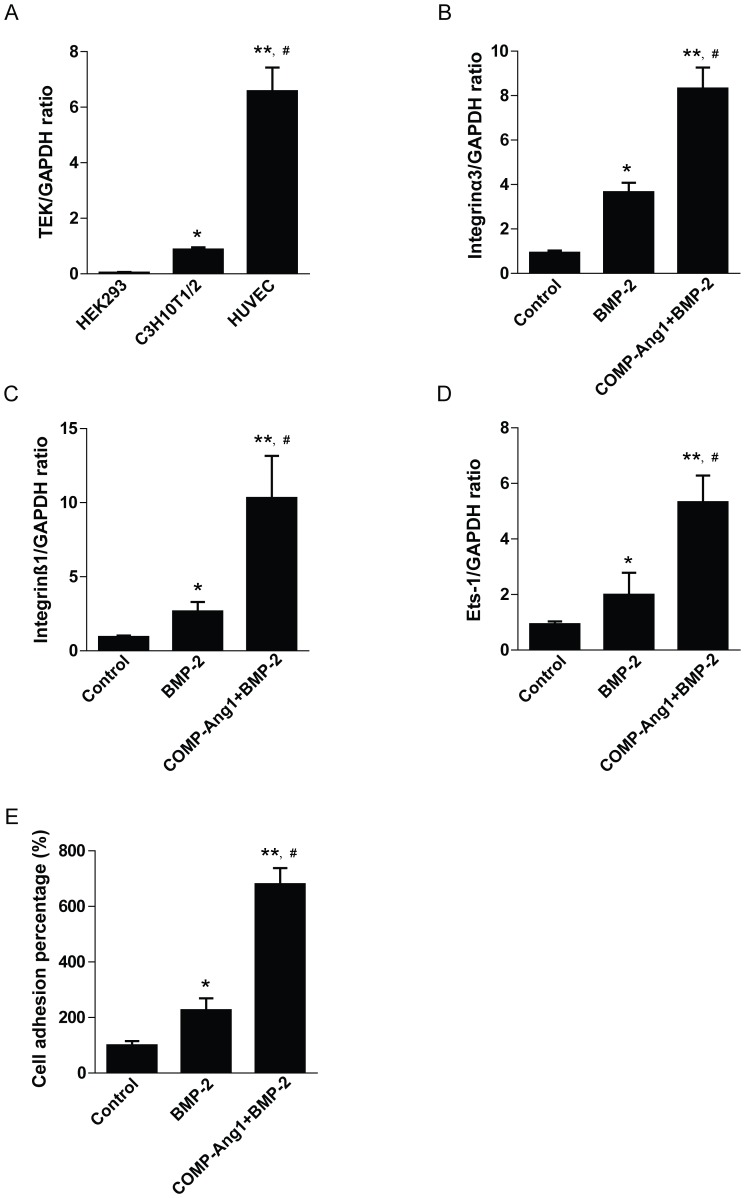
Expression of the Tie2 receptor and the effect of integrin on cell adhesion. (A) Endogenous expression of Tie2 in HEK293, C3H10T1/2, and HUVEC cells. *p<0.05 vs. HEK293; **p<0.01 vs. HEK293; ^#^p<0.05 vs. C3H10T1/2. (B–D) Real-time RT-PCR analyses for integrin α3, integrin β1, and Ets-1 were performed as described in the Materials and Methods. (E) Cell adhesion assays were performed in C3H10T1/2 cells after BMP-2 and COMP-Ang1 treatment. Values are the means ± SEM of three independent experiments. *p<0.05 vs. control; **p<0.01 vs. control; ^#^p<0.05 vs. BMP-2.

Generally, COMP-Ang1 and BMP-2 exert their influences by binding to receptors and stimulating subsequent target gene expression. Osteogenic differentiated C3H10T1/2 cells were evaluated by real-time RT-PCR and were shown to express the endogenous Tie2 receptor (Figure 8A). The concept of interactions between Ang1 and integrins is now gaining acceptance [19]. To test this, we performed real-time RT-PCR to determine integrin α3 and integrin β1 mRNA levels after the treatment of cells with COMP-Ang1 and BMP-2. Results demonstrated that integrin α3 mRNA expression level increased by 1.9- and 3.1-fold in the BMP-2 and COMP-Ang1 + BMP-2 groups, respectively, compared with that of the control group. Also, integrin β1 mRNA level increased by 1.9- and 3.9-fold in the BMP-2 and COMP-Ang1 + BMP-2 groups, respectively (Figures 8B and C). In addition, we observed increased mRNA levels of Ets-1, a transcriptional activator for α3 integrin [20], in the BMP-2 and COMP-Ang1 + BMP-2 groups. The mRNA level of Ets-1 was increased by 2.0- and 5.3-fold in the BMP-2 and COMP-Ang1 + BMP-2 groups, respectively, compared with that of the control group (Figure 8D). Finally, COMP-Ang1 and BMP-2 treatment promoted cell-to-cell adhesion (Figure 8E).

## Discussion

In our experiments, we examined the combined effect of COMP-Ang1 and BMP-2 in a rat model of surgery-induced necrosis of the femoral head. We found that combined intraosseous injection of COMP-Ang1 and BMP-2 effectively repaired ischemic damage by inducing angiogenesis and osteogenesis, and by decreasing osteoclast bone resorption activity. The rationale for the combined effect of these two proteins is based on three findings. First, COMP-Ang1 modulated bone repair by enhancing angiogenesis. Second, COMP-Ang1 enhanced BMP-2 signaling pathways to promote BMP-2 induced bone formation. Third, COMP-Ang1 enhanced cell adhesion and increased expression of Ets-1 and integrin α3β1 in osteoblast-differentiated C3H10T1/2 cells. To the best of our knowledge, the present study provides the first evidence that the combination of COMP-Ang1 and BMP-2 can be used to treat INFH.

Bone is a highly vascularized tissue and angiogenesis is crucial for bone regeneration. Neovascularization helps support the mesenchymal stem cells and osteoblasts necessary for bone repair [21]. Several studies using a bone fracture model have shown that osteogenesis is preceded by angiogenesis [22]–[24]. Thus, it is crucial to address the issue of angiogenesis in strategies for bone regeneration. *In vitro* studies have suggested that vascular endothelial growth factor (VEGF) and BMPs play important roles in cellular communication during angiogenesis and osteogenesis. Co-culture experiments have demonstrated that osteoblast-like cells stimulate the proliferation of endothelial cells through production of VEGF, whereas endothelial cells stimulate the differentiation of osteoprogenitor cells by the production of BMP-2 [25]. Furthermore, BMP-induced differentiation of preosteoblast-like cells enhanced the production of VEGF by the resulting osteoblasts [26], [27]. This BMP/VEGF interaction has also been demonstrated in bone regeneration studies where enhanced bone formation was shown when both growth factors were released or expressed simultaneously [28]–[30]. Patel et al. [31] investigated the dual delivery of VEGF and BMP-2 for bone regeneration in a rat cranial critical size defect model and found that dual delivery had synergistic effects and also resulted in faster healing times. However, the administration of exogenous VEGF often resulted in leaky, inflamed, and malformed vessels, which greatly compromised its therapeutic utility [32]–[34]. We used recombinant COMP-Ang1 protein as an angiogenic growth factor in this study. COMP-Ang1 has many potential advantages over the native Ang1, including efficiency of generating the recombinant protein, potency, and Tie2 activation. Furthermore, COMP-Ang1 can induce stable and functional blood vessel formation *in vivo* [14]. Upon COMP-Ang1 stimulation, Tie2 translocalization in endothelial cell–cell and cell–matrix contacts could be a main molecular event in the induction of angiogenesis and vascular enlargement [14], [35]. In an *in vivo* study, when COMP-Ang1 and BMP-2 were delivered simultaneously, more mature ectopic bones were formed than when BMP-2 was delivered alone [15]. Our *in vivo* results, derived from microCT and histology studies, revealed that bone regeneration was enhanced in the COMP-Ang1 + BMP-2 group as compared with that in the BMP-2 group.

In our previous study, we demonstrated that COMP-Ang1 accelerates the femoral head repair process [5]. The present study expanded this work to include the combination of COMP-Ang1 and BMP-2 in potentiating bone formation. BMP-2, a member of the TGF-β superfamily, plays a central role in osteoblast differentiation and bone formation through multiple downstream pathways, including the Smad pathway, which is initiated by the phosphorylation of Smad proteins by type I receptors, MAPK, and the PI3 kinase/Akt pathways [36]. The ultimate consequence of BMP-2 signaling is the activation of gene transcription, which promotes osteoblast differentiation and bone formation. Jeong et al. reported [15] that COMP-Ang1 enhanced the BMP-2-dependent transcriptional activity of the OG2 or 6xOSE promoters, indicating that the Ang1/Tie2 system might be involved in BMP-2-induced osteogenesis and modulation of BMP-2 target gene expression. They suggested that the effects of COMP-Ang1 on BMP2 activation of Smads, MAPK, and PI3-kinase/Akt signaling pathways may be a mechanism for the observed synergy. Tie2 phosphorylation followed by Ang1 binding to the receptor results in activation of PI3-kinase/Akt signaling pathway [37]. The present study showed that COMP-Ang1 stimulated BMP-2-mediated induction of Akt phosphorylation as well as Smads and p38 MAPK phosphorylation, indicating that the synergy of COMP-Ang1 and BMP2 on osteoblast differentiation may be related to at least partial overlap of signaling pathways in osteoblasts. The positive effect of BMP-2 on osteoblast cells is well known; however, high failure rates and complications were reported when rhBMP-2 was used in treatment [18], [38]. In addition, TGF-β is responsible for the therapeutic resistance of BMP-2, as it causes BMP signaling interference [38]. Transgenic mice lacking functional TGF-β signaling in osteoblasts [39] or mice treated with the TGF-β type I receptor kinase inhibitor SD-208 [40] have increased trabecular bone mass with tougher femurs and stiffer and stronger vertebral bodies. These data suggest that continuous exposure to active TGF-β might harm bone physiology, as can be seen in patients suffering from chronic inflammation, whose active TGF-β1 serum levels are often constantly elevated. In this study, serum TGF-β1 levels were increased 4-fold two weeks after surgery in all four animal groups (data not shown). One possible mechanism by which TGF-β1 may exert its inhibitory effect on osteoblast differentiation is through interference with BMP signaling. In this study, COMP-Ang1 decreased phosphorylation of Smad2/3, a downstream protein of TGF-β1 pathways. Therefore, these results suggest that COMP-Ang1 inhibits the TGF-β1 signaling pathway in addition to enhancing the BMP-2 signaling pathway.

According to previous studies, the Tie2 receptor, a target of COMP-Ang1, is expressed in quiescent hematopoietic stem cells of the bone marrow. Our study showed that the Tie2 receptor is expressed in C3H10T1/2 cells. Based on these findings, we hypothesized that COMP-Ang1 also plays a direct role in osteoblast differentiation through the Ang1-Tie2 pathway. Meanwhile, COMP-Ang1 interacts with integrins and promotes endothelial cell survival [17]. Integrins work as extracellular matrix receptors that transduce signals from the environment into the cell interior. Signals from integrin receptors regulate multiple cellular functions such as cytoskeletal organization and cell morphology, tissue-specific differentiation, cell proliferation, migration, and survival [41]. Zimmerman et al. reported impaired bone formation in transgenic mice having altered integrin function in osteoblasts [42]. Our results show that integrin α3 and integrin β1 mRNA levels were somewhat increased in the BMP-2 group and markedly increased in the COMP-Ang1 + BMP-2 group. In addition, we observed increased cell-to-cell adhesion in the COMP-Ang1 + BMP-2 group. These results suggest that COMP-Ang1 increases the expression of Ets-1 and its downstream integrin α3 and integrin β1 genes, which further increase cell-to-cell adhesion. Future studies are needed to provide direct evidence that COMP-Ang1 regulates the expression of integrins and subsequent cell-to-cell adhesion.

The dose of COMP-Ang1 utilized was 100 µg, which was determined to be sufficient to cure INFH in our previous study [5]. As we did not know how the osteonecrotic bone environment would influence the local bioavailability of BMP-2 or bone healing, the experimental doses of BMP-2 in this study were established based on our previous studies utilizing BMP-2 in a rodent model of spinal fusion [43]. The mean body mass, dose, and dose/weight assumed for humans are 70 kg, 1.5 mg, and 0.0214 mg/kg, respectively, resulting in values for the rat of 231.2 g, 4.93 mg, and 0.0214 mg/g, respectively. This led to the use of 5.0 g BMP-2 in rats.

There are some limitations of this study and its findings. One concern about the specific model used in this study is that revascularization is relatively robust in the rat model of ischemic necrosis. Another limitation of the study is our intraosseous injection method, which has the possibility for leakage and subsequent heterotopic ossification. The development of more precisely controlled release methods for direct intraosseous injection would help avoid this complication. Despite these possibilities, the combination of COMP-Ang1 and BMP-2 potentiated greater bone regeneration than was seen in the BMP-2 group. In summary, based on the findings of increased vascularity and new bone formation in ischemic femoral heads, a new strategy of COMP-Ang1 and BMP-2 combination therapy may be an ideal treatment for INFH.

## Supporting Information

Figure S1 **Cell culture and osteogenic induction of C3H10T1/2 cells.** (A) The expression levels of osteogenic genes (DMP1, BSP, and OC) and chondrogenic genes (aggrecan, Coll21a, and Col10a1) were assayed by real-time RT-PCR after BMP-2 treatment (200 ng/ml) for 14 days in osteogenic medium. (B) Matrix mineralization assessed by Alizarin red staining 2 weeks after incubation in osteogenic medium with rhBMP2 (200 ng/ml) or COMP-Ang1 (100 ng/ml) + rhBMP-2 (200 ng/ml). For the quantification of mineralization, the stained AR-S was eluted from the cell cultures with 10% cetylpyridinium chloride, and the dye concentration was measured via spectrophotometer. Data are expressed by mean ± SD. *p<0.05 vs. control; **p<0.01 vs. control; ^#^p<0.01 vs. BMP-2.
(TIF)
