# Supplementary figures and images for: COMP-Angiopoietin1 Potentiates the Effects of Bone Morphogenic Protein-2 on Ischemic Necrosis of the Femoral Head in Rats
Source: PLoS One. 2014 Oct 17;9(10):e110593. doi: 10.1371/journal.pone.0110593 (PMC4201557; doi:10.1371/journal.pone.0110593)

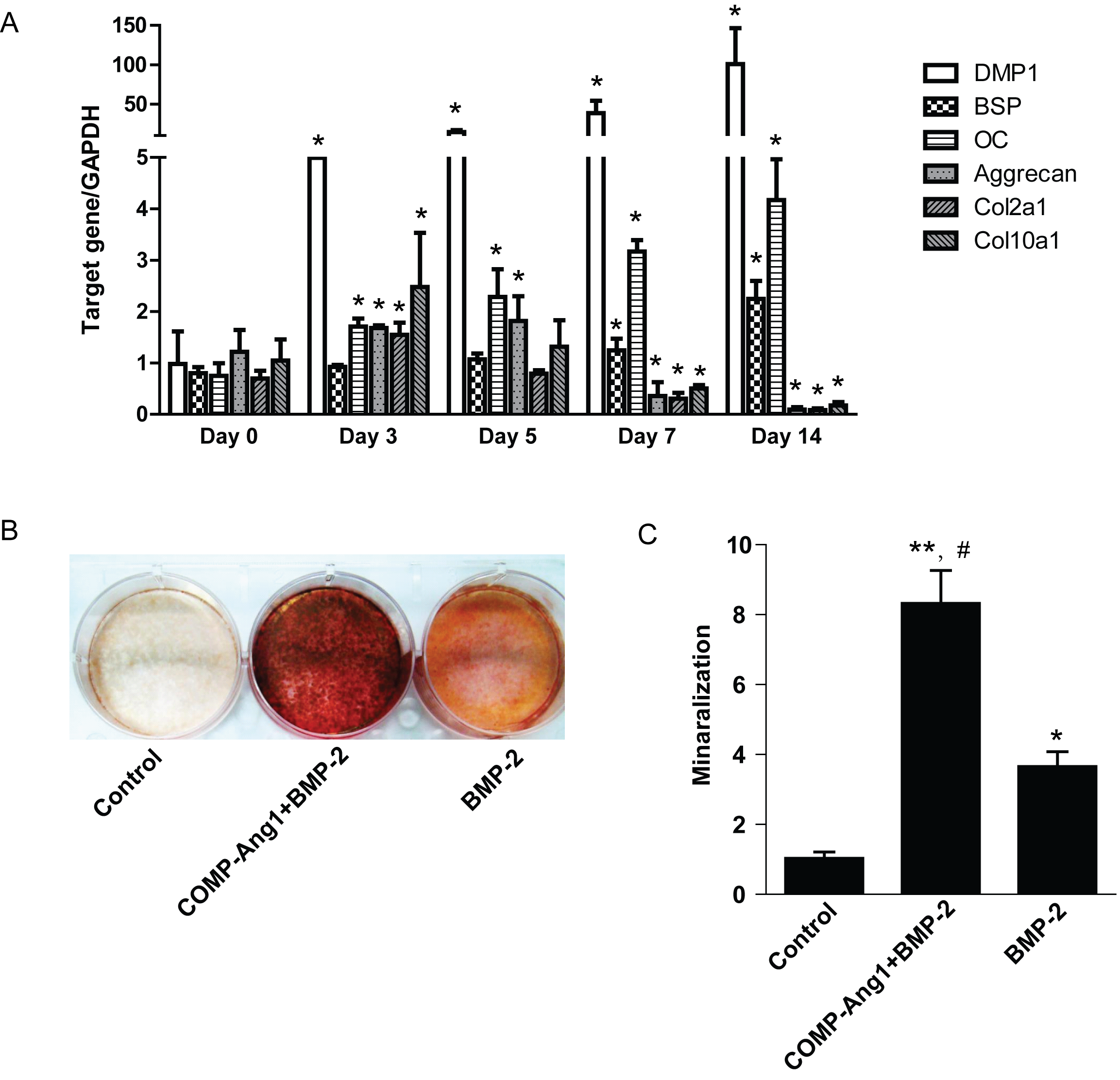

Supplement: Figure S1 — Cell culture and osteogenic induction of C3H10T1/2 cells. (A) The expression levels of osteogenic genes (DMP1, BSP, and OC) and chondrogenic genes (aggrecan, Coll21a, and Col10a1) were assayed by real-time RT-PCR after BMP-2 treatment (200 ng/ml) for 14 days in osteogenic medium. (B) Matrix mineralization assessed by Alizarin red staining 2 weeks after incubation in osteogenic medium with rhBMP2 (200 ng/ml) or COMP-Ang1 (100 ng/ml) + rhBMP-2 (200 ng/ml). For the quantification of mineralization, the stained AR-S was eluted from the cell cultures with 10% cetylpyridinium chloride, and the dye concentration was measured via spectrophotometer. Data are expressed by mean ± SD. *p<0.05 vs. control; **p<0.01 vs. control; #p<0.01 vs. BMP-2. (TIF) [file pone.0110593.s001.tif]
